# Development and Validation of the Content of a Mobile Digital Board-Game Application, Constituting a Playful Tool for the Management of Friction Injuries

**DOI:** 10.3390/nursrep16090305

**Published:** 2026-08-27

**Authors:** Rafael Henrique Marinho, Edmundo Martins Junior, Geraldo Magela Salomé

**Affiliations:** Professional Graduate Program in Applied Health Sciences, University of Sapucaí Valley, Pouso Alegre 37553-068, MG, Brazil; rafaelmarinho@univas.edu.br (R.H.M.); edmartinsjunior13@gmail.com (E.M.J.)

**Keywords:** friction, nursing assessment, wounds and injuries, nursing, game

## Abstract

**Objectives**: This study aimed to develop and validate the content of an educational board game aimed at training nurses in the management of friction injuries. **Methods**: For the development of the game, the Contextualized Instructional Design model was adopted, encompassing the phases of analysis, planning, development, and implementation. The validation of the Friction Care game content involved the participation of 50 expert nurses, who applied the Delphi Technique. Data analysis was conducted using the Content Validity Coefficient (CVC), with the aim of ensuring the consistency and relevance of the proposed items. **Results**: In the first evaluation round, the judges rated the game’s content as ranging from “inadequate” to “fully adequate.” After corrections based on the suggestions received, the material was resubmitted for analysis and then considered between “adequate” and “fully adequate.” The CVC ranged from 0.94 to 1.0 in the first evaluation and from 0.99 to 1.0 in the second, demonstrating high agreement among experts regarding the quality of the content. **Conclusions**: The game development and validation of the Friction Care game demonstrated its suitability as an educational resource for training nurses in the management of friction injuries.

## 1. Introduction

The integumentary system, the largest organ of the human body, performs vital protective and homeostatic functions. Its integrity may be compromised by intrinsic and extrinsic pathological factors, such as burns, traumatic ulcers, dermatitis, pressure injuries, and friction-related damage. These aggressions weaken the skin barrier and increase the risk of infections, electrolyte imbalances, and disturbances in thermal regulation [1,2].

Friction injuries (FIs) arise from mechanical forces applied to the skin, including friction, shear, or impact. Contact with rigid surfaces may lead to partial-thickness wounds characterized by separation between the epidermis and dermis—or full-thickness wounds, involving complete detachment of skin layers [3,4]. Studies indicate a higher prevalence of FIs in hospitalized older adults, particularly on the elbows (42%), legs (22%), and hands (13%), underscoring the vulnerability of these areas and the need for targeted preventive strategies [5,6].

The management of FIs requires a multidisciplinary approach that integrates physiological, anatomical, pharmacological, and educational measures aimed at prevention, healing, and reducing recurrence [6,7]. Despite their clinical relevance, research shows that many healthcare professionals, especially nurses, still present knowledge gaps regarding the etiology, prevention, and treatment of friction injuries, which may compromise care quality and patient safety.

In this context, educational technologies emerge as innovative tools to support clinical practice. Among them, educational games stand out for promoting active learning, stimulating decision-making, and reducing insecurity during the performance of procedures [2,7]. Despite their potential, there is a scarcity of pedagogical resources specifically designed to train professionals in the management of FIs, reinforcing the need for targeted educational strategies.

Educational games can assist in patient assessment, selection of dressings, implementation of preventive measures, treatment planning, and guidance for family members or caregivers. When grounded in scientific evidence, they contribute to health promotion and serve as support tools in moments of uncertainty, aiding daily clinical decision-making [2,7].

The development of the Friction Care game as a mobile application is justified because this format is particularly well suited to educational purposes that require active participation, repeated practice, and clinical reasoning. Unlike videos, which mainly deliver information passively, or distance-learning modules, which are often limited to reading and quizzes, an app-based game allows users to interact with realistic scenarios, make decisions, receive immediate feedback, and reflect on the consequences of their choices. In addition, the mobile format offers portability and accessibility, allowing users to access the content anytime and anywhere, which supports autonomous learning and reinforces content retention. Because the prevention and management of friction injuries involve judgment, problem-solving, and the application of knowledge to practice, the game-based application was chosen as the most appropriate educational strategy to meet these learning needs.

Thus, the proposed educational game represents an innovative resource developed with scientific foundations and media-based elements. Its purpose is to strengthen professional training by providing essential knowledge to reduce insecurity in clinical practice and enhance patient safety.

### Objective

This study aimed to develop and validate the content of an educational board game aimed at training nurses in the management of friction injuries.

## 2. Materials and Methods

This study is characterized as methodological research focused on the development and validation of a constructed health education technology [8].

The game’s design was based on the Contextualized Instructional Design approach, guided by constructivist principles. This strategy prioritizes the intentional planning of instructional situations, promoting educational actions integrated into the sociocognitive context of learners and encouraging meaningful knowledge construction [9].

The Contextualized Instructional Design Model directly aligns with the ADDIE model by structuring the development process into phases that correspond to analysis, design, development, and implementation. This alignment is not only conceptual but also methodological: both models organize instructional planning into sequential stages that ensure coherence between diagnosis, conception, production, and application. The key difference is that the contextualized model explicitly incorporates sociocultural and situational elements, expanding the traditional scope of ADDIE by emphasizing the relationship between content, context, and professional practice.

The adoption of this model is particularly relevant to the development of the game, as the project requires that the instructional content be integrated into scenarios, narratives, and challenges that reflect real work practices. The contextualized model provides a structure that guides this integration, ensuring that design decisions are not limited to the technical logic of the game but are aligned with educational objectives and the intended context of use.

Furthermore, the model contributes to ensuring consistency among objectives, mechanics, and assessment, since it guides the definition of learning goals from the analysis phase and ensures that each game element challenges, interactions, feedback, and performance criteria is derived from those goals. In this way, the development process maintains pedagogical alignment, preventing design decisions from being made in isolation or based solely on aesthetic or technological criteria. The result is a more coherent, intentional, and methodologically grounded educational product.

To ensure the effectiveness of the proposal, methodological guidelines structured into the stages of Analysis, Design, Development, Implementation, and Content Validation were adopted, as detailed below.

Stage 1—Analysis: The evidence review that supported the development of the game was conducted as an integrative review, beginning with the definition of the topic and the formulation of the guiding question: “What scientific evidence is available regarding the assessment, prevention, and treatment of friction injury?”

The question was structured using the PICO strategy, considering: Population—patients with risk factors for friction injury or those already presenting the injury; Intervention—clinical assessment, preventive measures, and treatment; Comparison—different interventions or absence of intervention; and Outcome—the effectiveness of interventions in assessment and care procedures [10,11].

Searches were conducted in February 2026 in the PubMed/MEDLINE, Cochrane Library, and SciELO Virtual Library databases, using descriptors in Portuguese, English, and Spanish, combined with the Boolean operators AND and OR. The search strategy included terms related to “friction,” “skin,” “Nursing Assessment,” and “Wounds and Injuries,” as well as their equivalents in other languages. Full articles published in peer-reviewed journals in Portuguese, English, or Spanish were included, focusing on the assessment of patients at risk for friction injury, preventive measures, and treatment. Studies that did not fit the thematic scope, documents without peer review, or publications without full-text access were excluded.

Study selection was performed independently by two authors through the screening of titles, abstracts, and subsequently full texts, with disagreements resolved by consensus. The search and selection process followed the PRISMA flowchart to ensure transparency, traceability, and reproducibility. After screening, eligible studies underwent data extraction and critical appraisal, considering author, year, country, study design, sample, main findings, and clinical implications for the development of the game.

The methodological quality and level of evidence of the included studies were classified according to the Agency for Healthcare Research and Quality (AHRQ), whose hierarchy comprises six levels: level 1—evidence from meta-analyses of multiple controlled and randomized clinical trials; level 2—individual experimental studies; level 3—quasi-experimental studies; level 4—descriptive, correlational, qualitative, or case studies; level 5—case reports or systematic program evaluations; and level 6—expert opinion. For this study, evidence with greater methodological robustness and clinical relevance was prioritized, and only studies with an evidence level above 4 were included in the synthesis that supported the game’s content.

The synthesis of findings allowed the identification of key scientific elements necessary to compose the educational content of the game, particularly those related to the definition of friction injury; clinical assessment; risk factors; cleaning; classification into categories I, II, and III; preventive measures; and therapeutic approaches. Thus, the game was developed based on updated evidence, selected systematically and rigorously, strengthening its scientific validity and relevance for clinical practice [12].

In addition to classification according to level of evidence, various relevant information was collected from the studies included in the review. Extracted data included: title of the publication, year of publication, country of origin, language of the article, research objectives, methodological design adopted, main findings, conclusions presented by the authors, and detailed description of the interventions analyzed. This systematization allowed a comparative analysis among the studies, contributing to the construction of a critical and well-founded synthesis on the investigated topic.

The information systematized from the selected studies was fundamental to support a critical and in-depth analysis of the available scientific evidence on the topic. This approach made it possible to identify knowledge gaps, recognize effective clinical practices, and point out promising directions for future investigations focused on the assessment, prevention, and treatment of FIs.

Stage 2—Design: This phase consisted of the development of the instructional materials used in the educational game Friction Care. The themes and topics to be addressed were defined based on the findings of the integrative literature review, which identified the main scientific evidence related to the assessment, prevention, and treatment of FIs. Based on this evidence, explanatory texts were developed, priority content was selected, and supporting visual elements were created.

Furthermore, the most appropriate media formats were chosen, and the layout of the activities was designed to integrate the review findings with constructivist pedagogical strategies. The content was organized into sequential topics to facilitate assimilation, promote meaningful learning, and make the educational process more interactive and engaging.

Topic 1—Clinical Assessment: In this initial stage, the educational game Friction Care presents tools for the detailed analysis of the patient’s skin conditions. Risk factors for the development of FI are considered, as well as characteristics such as edema, coloration, presence of ecchymosis, integrity of the skin flap, changes in tone (pallor, opacity, or darkening), bleeding, type of tissue, exudate, and lesion dimensions [13,14,15].

Topic 2—Injury Classification: The Brazilian version of the Skin Tear Classification System (STAR) is used [16,17,18], which includes three categories of injury:

- Type I: No skin or flap loss, allowing repositioning during dressing.
- Type II: Partial flap loss, preventing coverage of the wound.
- Type III: Total absence of the flap, leaving the wound completely exposed.

Topic 3—Preventive Care: Practices are established to prevent injuries from worsening, such as the use of hypoallergenic moisturizers, appropriate mobilization and transfer techniques, repositioning, care with adhesives, and hygiene with neutral soaps or those containing aloe vera. The use of alkaline, antibacterial, or perfumed products is contraindicated [19,20,21].

Topic 4—Therapeutic Management: The clinical management of the injury is described, including local skin care and the selection of the ideal dressing. This should facilitate healing, reduce pain, act as a barrier against bacterial infections, and allow atraumatic removal [22,23,24].

Stage 3—Development: This stage comprised the selection of the tools that composed the phases and spaces of each stage of the Friction Care game, the definition of the navigation structure, and the planning of the environment configuration.

Stage 4—Implementation: In this phase, the technical configurations of the tools and digital educational resources that compose the game were defined. A virtual environment was also developed to make the application available, allowing users to download it via the internet and install it on mobile devices.

Stage 5—Validation of the game content: Friction Care—After the game’s development, validation was initiated with 50 nurse judges. For the sample calculation, the formula for an infinite population was used, where $n=\frac{Z_{1-\alpha /2}^{2}\cdot P(1-P)}{e^{2}}$, in which $Z_{1-\alpha /2}$ refers to the adopted confidence level (95%); P represents the expected proportion of experts (80%), indicating the adequacy of each item; and “e” represents the acceptable difference in proportion in relation to what would be expected (15%), resulting in a minimum sample of 27 professionals.

The inclusion criteria for evaluators were: professionals holding a bachelor’s degree in nursing, with at least two years of experience. The exclusion criteria were evaluators who agreed to participate in the research but did not respond and/or submit the evaluation questionnaire within 15 days.

Recruitment was carried out through direct contact via institutional email, inviting professionals who met the eligibility criteria. Experts were considered eligible if they were nurses with a degree in nursing, specialization in stomatherapy or dermatology, at least two years of experience in the field, and documented involvement in clinical practice, teaching, or research related to educational technology. In addition, priority was given to professionals who were members of wound care committees, patient safety groups, or who had scientific production in the area. This identification and selection strategy sought to ensure institutional diversity, practical experience, and technical qualification, strengthening the credibility of the expert panel involved in the evaluation of the technology.

To ensure that the evaluators had sufficient experience, technical knowledge, and practical background to contribute meaningfully to the consensus process, the profile of the specialists was defined beforehand, considering academic training, years of professional practice, clinical or teaching experience, scientific output, and involvement in activities related to the topic. The selection was intentional, directed toward professionals recognized for their expertise. After identifying potential participants, a formal invitation was sent, presenting the study objectives, the role of the experts, the anonymous nature of the responses, and the flow of the evaluation rounds.

In the first round, the experts received the material to be analyzed and performed an individual evaluation, assigning scores and providing qualitative comments. The responses were compiled and analyzed by the research team, which identified items with low agreement, high variability, or relevant suggestions for improvement. These items were revised and sent back to the experts in the second round, accompanied by a summary of the group’s contributions, while maintaining anonymity. The participants then re-evaluated the items in light of the collective feedback, allowing for deeper reflection and adjustment of opinions. The process could continue for additional rounds until response stability was observed, characterized by convergence of evaluations and reduced variability. This flow ensured that the final consensus was robust, technically grounded, and methodologically consistent.

Each evaluator received, by email, an invitation letter containing: institutional and personal presentation; contextualization of the research; Research Ethics Committee approval; clarification regarding the relevance of the specialists’ contribution; and detailed instructions for participation.

The invited participants were duly informed about the study’s objectives, procedures, and nature. Those who agreed to participate formalized their consent by signing the Free and Informed Consent Term (FICT), in accordance with current ethical guidelines. The deadline established for submitting responses was 15 days after receipt of the invitation.

The assessment instrument was structured in two sections. Section 1 addressed aspects related to the game’s content (questions related to friction injury) (8 items), corresponding specifically to the following topics: definition of friction injury; clinical assessment and identification of risk factors for friction injury in patients; cleaning of the friction injury; category I friction injury; category II friction injury; category III friction injury; preventive measures for friction injury; and treatment according to the classification of the friction injury.

Section 2 dealt with functionality, usability, and efficiency aspects (14 items), specifically covering: Functionality—correct execution of the game’s functions; relevance of content to the target audience; presence of functions necessary for assessment, prevention, and treatment of friction injury; and secure access via password. Usability—clarity of instructions; ability of the content to facilitate teaching and learning; ease of learning to use the game; control over actions and ability to undo errors; and clear and concise tutorial. Efficiency—vocabulary accessible to the target audience; appropriate response time; maintenance of player motivation; application stability, loading speed, and performance; and suitability of the resources provided.

Response scale and validity criteria: Responses were recorded on a five-point Likert scale with the options “not applicable,” “inadequate,” “partially adequate,” “adequate,” and “totally adequate.” For the quantitative content validation analysis, responses classified as “adequate” (3) or “totally adequate” (4) were considered valid. The options “partially adequate,” “inadequate,” and “not applicable” were treated as indicators of the need for revision.

Analysis procedure: The analysis included two approaches. First, a quantitative analysis of the frequencies of the “adequate” and “totally adequate” categories per item to identify items with favorable consensus. Second, a qualitative analysis of the open comments provided by the experts to identify specific problems, ambiguities, omissions, and suggestions for improvement in both the clinical content and the interface and performance features.

The evaluators used the Delphi Technique to assess the content as well as the functionality, usability, and efficiency of the material. The Delphi Technique is a structured consensus method that gathers expert opinions through successive rounds of evaluation. Its central principle is obtaining qualified judgments anonymously, reducing influence bias and allowing each participant to assess the items independently. In each round, experts assign scores, analyze the content, and provide qualitative comments. The responses are then compiled and returned to the group with anonymity preserved, which encourages more impartial and well-grounded reflections. This iterative process makes it possible to identify divergences, adjust content, and promote progressive refinements until stability in the responses is achieved [25].

The repetition of rounds is essential for transforming an initial agreement—often merely numerical—into a qualified consensus that integrates both quantitative metrics and qualitative analyses. By allowing experts to reconsider their evaluations in light of collective feedback, the Delphi Technique enhances methodological rigor, strengthens conceptual precision, and ensures that the final product truly reflects the synthesis of the evaluators’ technical expertise and judgment. For this reason, the method is widely used in studies involving the construction, validation, or refinement of instruments, protocols, and educational materials, especially in complex areas or those with limited evidence [25].

The criteria for including items in the second round of the Delphi Technique were:

- CVC below 0.78, indicating the absence of an acceptable minimum level of consensus.
- CVC ≥ 0.78, but with instability in the responses, evidenced by high variability in the ratings among evaluators.
- High CVC, including values > 0.90, but accompanied by qualitative disagreements, such as comments pointing to doubts, ambiguities, or the need for conceptual adjustments.
- Relevant suggestions from experts indicating that the item could be improved for greater clarity, technical precision, or clinical applicability.
- Heterogeneity in the evaluations, even with a satisfactory CVC, demonstrating the absence of consolidated consensus.

Statistical analysis was conducted using the Content Validity Coefficient (CVC), adopting a minimum acceptance criterion of 0.70 [8,26]. The CVC calculation followed systematized steps: The mean score assigned to each item (M) was obtained. This value was then divided by the maximum value of the scale used (Vmax), resulting in the initial CVC (CVCi). To increase the precision of the measure, a correction for the evaluators’ judgment error (Pej), derived from the variability of the responses, was applied. The final coefficient (CVCc) was then calculated using the formula:

$$CVC_{c}=CVC_{i}-Pej$$

Items that did not reach the minimum established value were reformulated based on the specialists’ suggestions and submitted to a new evaluation round, ensuring greater rigor, clarity, and reliability in the content validation process.

## 3. Results

**Figure 1:**
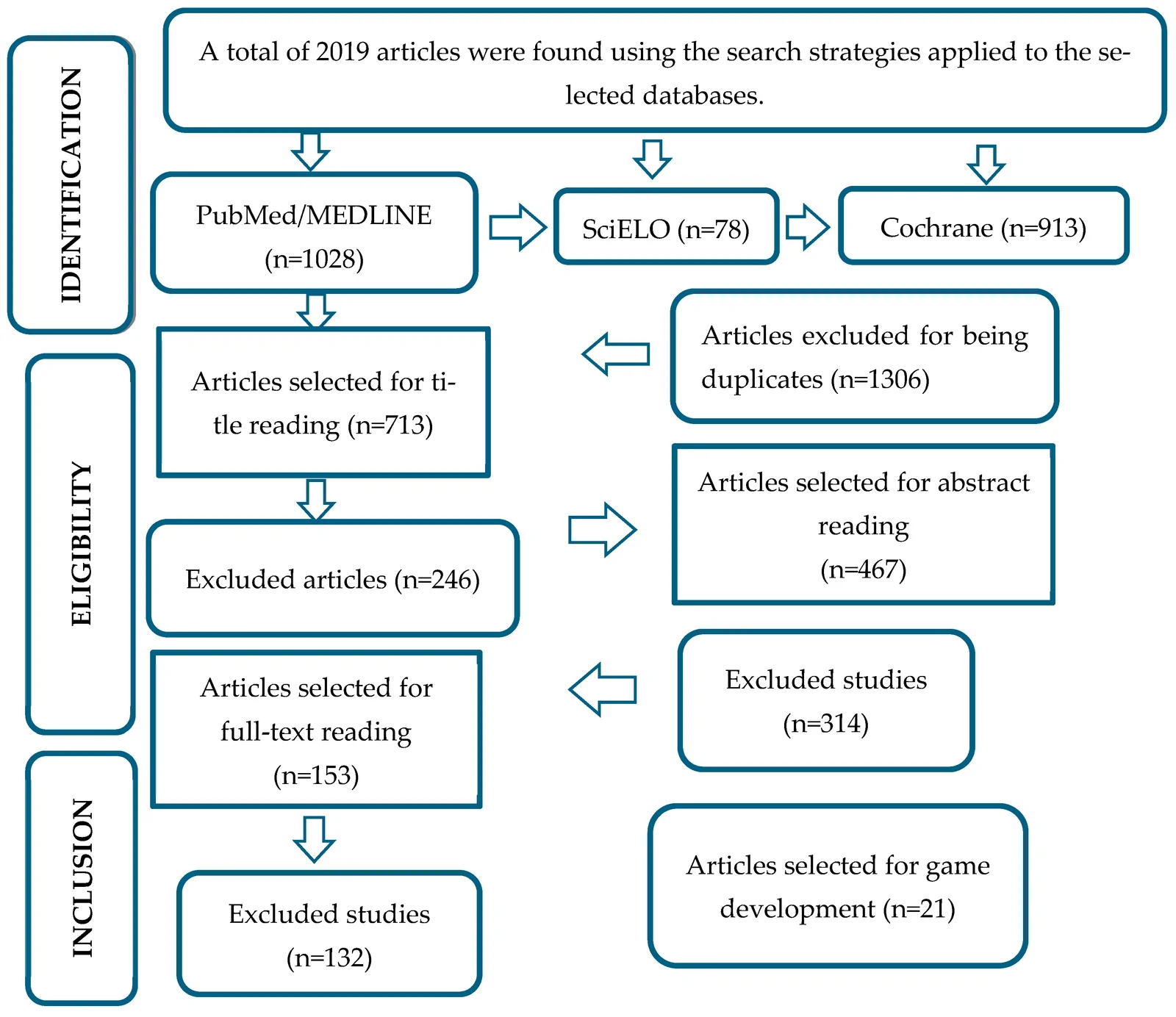
Mapping and selection stages of the studies used in the educational game development on friction injury.

During the integrative literature review process 2019 publications were initially identified. After the removal of 1306 duplicate records 713 studies remained for title screening. Of these, 467 were selected for abstract reading, resulting in 153 articles submitted to full-text review. At the end of the analysis, 21 studies met the established criteria and were used as the basis for the development of the educational game Friction Care (Figure 1).

Table 1 presents the articles selected during the integrative literature review that guided the development of the present game. A total of 21 articles were included, classified according to their level of evidence. Among them, four articles had level I evidence, eight articles had level II evidence, four articles had level III evidence, and five articles had level IV evidence.

Table 2 presents the suggestions made by the judges during the evaluation of the content of the Friction Care game and the changes implemented.

**Figure 2:**
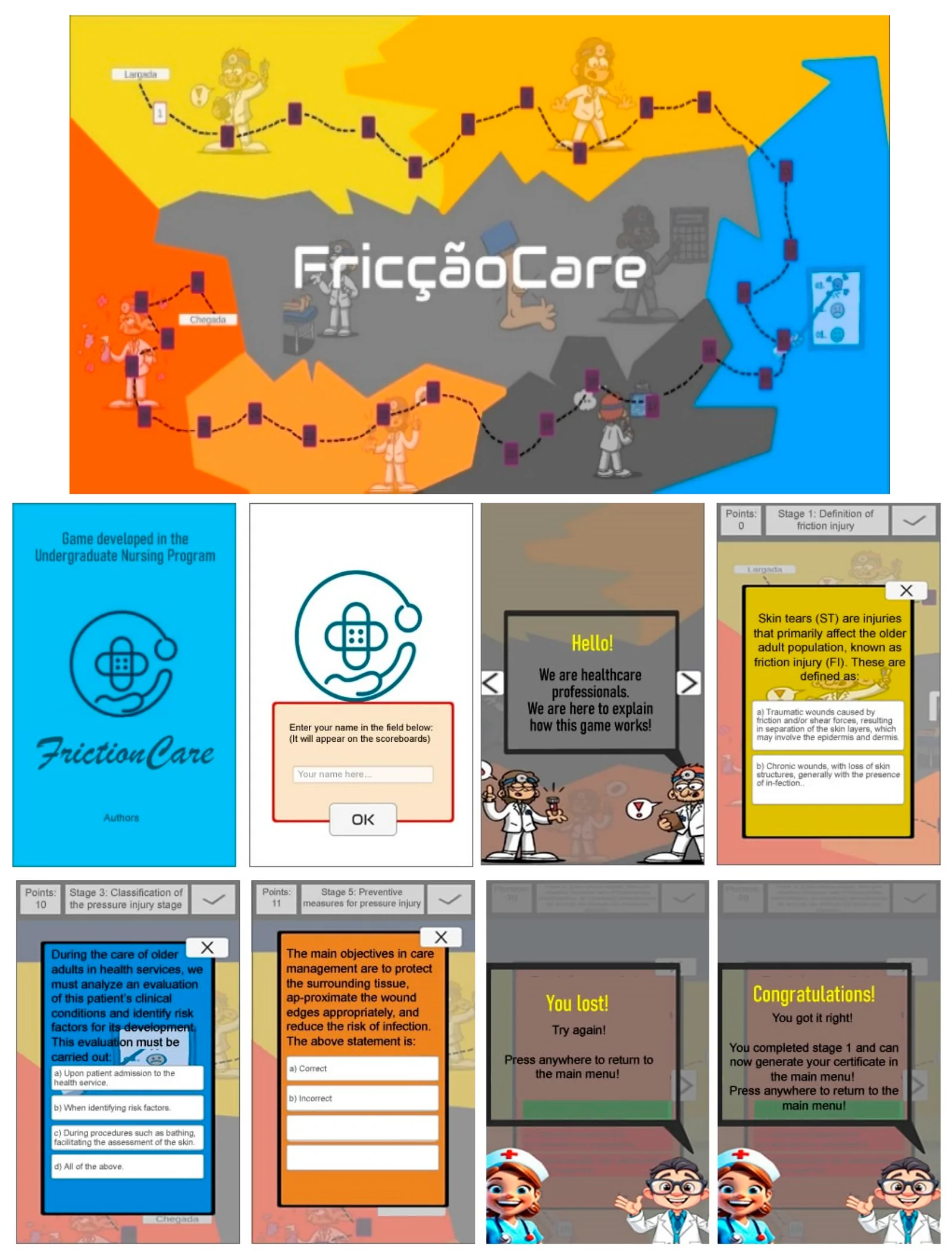
Board and some cards with questions from the Friction Care game.

Figure 2 shows some screenshots of the game, which is publicly available through a shared link on Google Drive: https://drive.google.com/file/d/1D0eKghhwLzTVMPxGlGKo3HvJyBV3VHP2/view?usp=sharing (accessed on 10 December 2025).

Players answered 90 multiple-choice questions presented on cards distributed across a virtual game board. These questions were developed by the authors themselves, using the references corresponding to each topic included in Stage 2—Design. In the Appendix A, we included some questions that users answered when playing the game (Appendix A).

Because it is an online game, the board does not have physical dimensions; it was designed as a responsive rectangular layout that automatically adjusts to the resolution of different devices (smartphones and tablets). On the interface, it occupies approximately 70% of the screen width and is divided into nine stages, each containing 10 spaces, forming a path from “start” to “finish”.

When starting the game, the user sees the game’s title, the authors’ identification, and the institution where it was developed. By swiping the screen to the right, the option to create a password appears, allowing the player’s progress to be saved. Then, by clicking Play, a tutorial is displayed across five explanatory screens. After this stage, the board appears on the main screen.

At the top margin, two indicators are shown: the first displays the accumulated score, and the second shows the total number of questions answered. By clicking the side arrow, the card containing the question is presented. The player selects an alternative and, upon confirming the answer, the system automatically records whether it is correct or incorrect. Correct answers add points, while incorrect answers do not generate points but allow the user to proceed to the next question. The scoring system is cumulative and updated in real time.

The game incorporates an immediate feedback mechanism: after each answer, the system informs the user whether the chosen alternative is correct, reinforcing learning and promoting self-correction. This feature was included as part of the pedagogical validation. The overall architecture of the game consists of the following modules:

1. Authentication (password creation and player identification) and Tutorial (five explanatory screens).
2. Virtual board (nine phases and 90 cards).
3. Question module (display, answer selection, and feedback).
4. Scoring module (automatic calculation and display).
5. Progress tracking (local storage linked to the password).

The Friction Care game was registered with the National Institute of Industrial Property under number BR512024004801-4.

Of the 50 participating judges, 20% had between 2 and 5 years of professional experience, 46% had between 6 and 10 years of experience in the field, and 34% had more than 11 years of experience. Regarding specialization, 64% were stomatherapists and 36% worked in the area of dermatology. In terms of academic background, 30% were specialists, 40% held a master’s degree, and 30% held a doctoral degree.

The geographic distribution of the judges showed that 20% were from the North region, 20% from the Northeast, 16% from the Center-West, 26% from the South, and 18% from the Southeast.

Table 3 presents the questions submitted for evaluation by the professionals, along with the respective CVC values. In the first round of evaluation, the indices ranged from 0.94 to 1.0; in the second round, they ranged from 0.99 to 1.0. These results indicate a high level of agreement among the evaluators, demonstrating the excellence of the content developed for the educational Friction Care game. The time interval between the first and second rounds was 15 days each. Thus, 15 days were required for the first round and another 15 days for the second, totaling 30 days for the complete evaluation of the game.

Table 4 details the main topics addressed by each item of the applied questionnaire, as well as the qualitative evaluation conducted by the specialists regarding the content of the Friction Care game, using the Delphi Technique. In the first round of analysis, the opinions ranged from inadequate to totally adequate. After a second round of evaluation, consensus was observed among the judges regarding the relevance and adequacy of the game.

## 4. Discussion

The development of educational technologies in nursing represents an innovative strategy to enhance clinical practice and promote safe care grounded in scientific evidence. Resources such as applications, games, booklets, and digital platforms have been widely created and validated, becoming tools incorporated into daily clinical practice and professional training [47,48].

In this study, the Friction Care educational game was developed to support teaching related to the care of patients with risk factors for, or already presenting, friction injury (FI). The content was structured based on an integrative literature review, with studies classified according to their level of evidence and subsequently validated by experts in the field. The evaluators were nurses working in both higher education institutions and hospital services, which allowed the integration of perspectives from professionals involved in academic training and those engaged in daily clinical practice. This intentional composition strengthened the robustness and pedagogical applicability of the material.

The second round of the Delphi Technique was essential to reinforce the scientific validity, clinical relevance, and technical robustness of the game’s content, even with CVC values above 0.90 in the first round. Although high scores indicate numerical consensus, they do not capture conceptual nuances, interpretative divergences, or instabilities in the responses. The analysis of experts’ comments and suggestions revealed ambiguities and areas for improvement that were not evident in the quantitative indicators, allowing adjustments that enhanced the precision and technical coherence of the content [39].

Furthermore, divergences among experts often reflect real challenges in clinical practice, such as differing interpretations of educational materials (apps, games, booklets) or specific needs in particular care settings. Revising the items based on collective feedback and submitting them to a new evaluation round made it possible to consolidate a more robust consensus, transforming statistical agreement into qualified consensus and increasing the safety, usefulness, and relevance of the material for professional practice [49].

The results reinforce the importance of innovative educational strategies for strengthening health education. Educational technologies help prepare professionals who are more critical, confident, and attentive to the prevention of skin injuries, especially among vulnerable populations such as older adults. Friction Care stands out by offering an active learning environment that stimulates essential competencies such as clinical reasoning, decision-making, and teamwork. Its playful and interactive dynamics complement traditional teaching methods, promoting greater engagement and supporting knowledge consolidation through the simulation of clinical situations in a safe environment.

The construction of scenarios based on real and updated professional practice enhances students’ analytical capacity and fosters autonomy. The accessible design and intuitive interface make the resource inclusive, even for users with limited familiarity with digital games, expanding its potential use across diverse educational contexts. Evidence already demonstrates that educational games can improve engagement, knowledge retention, and the development of practical competencies in nursing. In the case of Friction Care, the use of realistic clinical scenarios with multiple possible outcomes contributes to the development of autonomy and critical thinking when facing the complexity of care [49].

Furthermore, educational technologies play an important role in bridging theory and practice by offering simulated environments in which error becomes part of the learning process. This characteristic helps prepare future professionals more effectively, reduces care-related risks, and strengthens patient safety [7,49]. Integrating friction injury care into undergraduate nursing education broadens the scope of health training by promoting not only technical knowledge but also proactive attitudes in risk situations such as FI. Thus, this educational technology not only innovates but also contributes to evidence-based and patient-centered practices.

Recognizing the limitations of the study, the manuscript was revised to clearly state that the investigation is restricted exclusively to content validation, without assessing educational effectiveness, usability, user satisfaction, or clinical impact. These aspects were not part of the methodological scope and remain directions for future research. This study evaluated only the content validity of the educational material. No tests were conducted in real-world settings with students or practicing professionals, nor were assessments performed to determine educational effectiveness or clinical impact. Therefore, it is not possible to measure the game’s influence on learning outcomes or care practices. These aspects will be explored in subsequent studies, including an upcoming investigation to be conducted by a master’s student at the institution, in which these practical evaluations will be systematically addressed.

## 5. Conclusions

Friction Care is an educational technology designed to support continuing nursing education, integrating technical content with playful elements. The game was developed based on scientific evidence and validated by specialists in terms of content, functionality, usability, and efficiency. Therefore, Friction Care may be considered a promising educational resource in nursing, especially for supporting learning about the assessment, prevention, and treatment of friction injury.

## 6. Patents

Software registration at the National Institute of Industrial Property (Brazil).

## Figures and Tables

**Table 1:** Summary of the articles included in the integrative literature review.

Author(s) Title Journal/Year/Volume/Issue	Abstract	Level of Evidence
Ceyhan Ö, Kaçmaz HY, Ozen B, Aslaner H, Ökdem AF. “Skin tears in older adults people receiving home care.” *BMC Geriatr*. 2025 26(1): 147. doi: 10.1186/s12877-025-06769-w [27]	The prospective cohort study evaluated 161 older adults receiving home health services with the aim of identifying the prevalence of skin lacerations. Data were collected using validated clinical instruments, including the Skin Laceration Scale, the Mini Nutritional Assessment, the Braden Scale, and AVPU. The sample had a mean age of 77 years and was 51.6% female. Twenty-three percent were found to have pressure ulcers, predominantly stage 1 and located on the feet. Reduced skin turgor and absence of cutaneous bleeding showed a significant association with the presence or absence of these lesions. Changes in skin coloration and difficulty ambulating were statistically significant predictors of pressure injury development. The authors concluded that physiological changes related to aging influence the occurrence of skin lesions, underscoring the need for preventive strategies in home care.	04
Holloway S et al. “Development and Validation of the International Skin Tear Advisory Panel Skin Tear Data Collection Tool.” *Adv Skin Wound Care*. 2025. 38(10): 527–533. doi:10.1097/ASW.0000000000000372 [28]	The authors validated a data collection instrument for skin lacerations. Fifteen experts participated, assessing the instrument’s face validity and providing suggestions for improvement. The final version of the tool comprises 22 items addressing patient demographics, clinical characteristics of the skin laceration, associated risk factors, and contextual information. An international panel of experts reached consensus after a qualitative review conducted in two rounds, resulting in subsequent adjustments to the instrument. The ISTAP DC tool was developed based on evidence-based recommendations and validated by an international expert panel. Its implementation will assist healthcare professionals and researchers in the standardized collection of epidemiological data, contributing to improvements in clinical practice, quality initiatives, and future research focused on the prevention and treatment of skin lacerations.	02
Jiang Q, Wan L, He H, Fu X, Lan S, Yang Y, He F, He M. “Development and Psychometric Evaluation of KAP-ST: A Knowledge, Attitude and Practice Instrument for Care Workers at Preventing Skin Tears.” *J Adv Nurs*. 2026; 82(5): 5359–5375. doi: 10.1111/jan.70149 [29]	The study developed and psychometrically evaluated the KAP-ST instrument, designed to assess caregivers’ knowledge, attitudes, and practices regarding the prevention of skin tears in older adults. The development process occurred in four phases, including a comprehensive literature review, expert validation using the Delphi method, item optimization through a cross-sectional study, and final psychometric testing with long-term care professionals. The final instrument contains 35 items across three dimensions and demonstrated excellent content validity, strong factor structure, and high reliability. The KAP-ST is suitable for assessing caregivers in the prevention of skin tears in older adults, although further research is needed to refine and confirm its validity.	02
Mornane C et al. “Twice-Daily Moisturizer Application for Skin Tear Prevention among Older Adults in Acute Care.” Advances in Skin & Wound Care, 2021; 34(2). DOI: 10.1097/01.ASW.0000725180.14180.da [30]	Evaluates twice-daily neutral pH moisturizer as prevention for skin tears. Results were unsatisfactory due to care inconsistencies and lack of product availability.	02
Cilluffo S, Bassola B, Beeckman D, Lusignani M. “Risk of skin tears associated with nursing interventions: a systematic review.” J Tissue Viability. 2023; 32(1): 120–129. doi: 10.1016/j.jtv.2022.11.006. [31]	This systematic review examined nursing interventions that increase the risk of skin tears in older adults. Seventeen studies published between 2012 and 2022 were included. Practices such as cleansing with cold water and soap, not applying moisturizing or protective products, wearing short sleeves, abrupt patient transfers, wearing jewelry or having long nails, and improper removal of adhesive dressings were associated with a higher risk of skin tears. The review concludes that nursing staff must understand which interventions place patients at risk and which practices are recommended to prevent these injuries, as nursing care directly affects skin integrity.	01
Hickey T, Ayres J. “Who should be the first responders for the management of skin tears?” Journal of Wound Care, *2021*; 30(5). DOI: 10.12968/jowc.2021.30.5.332 [32]	Study in an ambulance service in eastern England identifying significant numbers of community skin tears. Highlights need for prevention in home care, transport, long-term care, and hospitals.	03
LeBlanc K, Woo K. “A pragmatic randomised controlled clinical study to evaluate the use of silicone dressings for the treatment of skin tears.” International Wound Journal, 2022; 19(1). DOI: 10.1111/iwj.13604 [33]	Canadian randomized study evaluating silicone dressings. “Mepilex Border Flex” showed high effectiveness, healing in 7–21 days, faster than standard silicone dressings (>3 weeks).	02
Al Khaleefa N et al. “What is the impact of skincare bundles on the development of skin tears in older adults? A systematic review.” International Journal of Older People Nursing, 2022; 17(4). DOI: 10.1111/opn. 12455 [23]	Addresses risk factors such as dry/thin skin and functional dependence. Emphasizes implementing care bundles and investing in nursing education.	01
Kaçmaz HY et al. “The prevalence and factors associated with skin tears in hospitalized older adults: A point prevalence study.” Journal of Tissue Viability, 2022; 31(3). DOI: 10.1016/j.jtv.2022.06.001 [34]	Study in Turkey assessing prevalence of skin tears. Highlights need for holistic care, especially for older adults.	04
Holloway S et al. “Reapproximating a Skin Tear Flap.” Advances in Skin & Wound Care, 2022; 35(8). DOI: 10.1097/01.ASW.0000835124.90642.ce [35]	Brief study identifying risk factors: general health, mobility, and skin problems. Recommends immediate measures such as calcium alginate dressings and flap repositioning.	03
Chou SP et al. “Multi-disciplinary management of type 1 and 2 skin tears using a silver-based hydrofiber dressing.” Medicine, 2023; 102(37). DOI: 10.1097/MD.0000000000035112 [36]	Lists recommended dressings and evaluates silver hydrofiber dressing as first-line treatment for type 1 and 2 skin tears.	04
Kacmaz HY et al. “Skin properties associated with skin tears in older adults: A case-control study.” Journal of Tissue Viability, 2023; 32(4). DOI: 10.1016/j.jtv.2023.09.004 [37]	Study in long-term care and elderly daycare centers identifying skin types susceptible to skin tears. Highlights impact on quality of life, hospitalization time, and healthcare costs.	01
Fulbrock RN P et al. “Healing rate of hospital-acquired skin tears using adhesive silicone foam versus meshed silicone interface dressings: A prospective, randomized, non-inferiority pilot study.” International Journal of Nursing Practice, 2023; 30(3). DOI: 10.1111/ijn.13229 [38]	Compares adhesive silicone foam vs. meshed silicone interface dressings. Both resulted in complete healing in <21 days.	03
Pinheiro RV et al. “Algorithms for prevention and treatment of friction injury.” Acta Paulista de Enfermagem, 2021; 34. DOI: 10.37689/acta-ape/2021AO03012 [39]	Develop and validate the content of algorithms for the prevention and treatment of friction injury. For the construction of algorithms, an integrative literature review was conducted after searching SciELO, LILACS and MEDLINE databases. The algorithms were evaluated by 26 raters—10 physicians and 16 nurses—using the Delphi Technique. The results were analyzed by the Content Validity Index (CVI). In the first evaluation cycle, the items of the algorithms were considered “inadequate” to “totally adequate” by the raters. After adjustments suggested by the raters, the algorithms were submitted to a second evaluation cycle, when all items were considered “adequate” or “totally adequate,” resulting in a content validity index of 1.0. The algorithms have valid content and can help health professionals in the evaluation, prevention and treatment of friction injuries.	04
Fan S, Jiang H, Shen J, Lin H, Yang L, Yu D, Zhang M, Zheng N, Chen L. “Prediction models for skin injuries in older adults: a systematic review and meta-analysis.” Geriatr Nurs. 2024 Sep–Oct; 59: 103–112. doi: 10.1016/j.gerinurse.2024.06.030 [40]	This systematic review and meta-analysis evaluated published prediction models for skin-injury risk in older adults. A comprehensive search across international and Chinese databases identified 1499 studies, from which eight prediction models were included. All studies used logistic regression, with reported skin-injury prevalence ranging from 3% to 33.3%. Senile purpura and a history of previous skin injuries were the most common predictors. Model performance was moderate (AUC 0.765–0.854), but all studies showed a high risk of bias, particularly in outcome and analysis domains, and four studies presented serious concerns regarding applicability. The review concludes that future research should focus on developing new prediction models with larger samples, stronger study designs, and external validation from multiple sources to improve accuracy and clinical usefulness.	01
Da Silva, Glebia Queiroz et al. “Skin care for older adults: prevention of friction injuries and xerosis.” Revista Foco (Interdisciplinary Studies Journal). 2026; 19(5):1. doi: 10.54751/revistafoco.v19n5-098 [41]	This integrative literature review analyzed nursing care aimed at preventing xerosis cutis and friction injuries in older adults. A search across national and international databases identified 23 studies published between 2018 and 2025. The findings revealed a high prevalence of these skin conditions, particularly among hospitalized or institutionalized older adults, due to skin fragility, immobility, comorbidities, and functional dependence. Xerosis cutis was identified as an important predisposing factor for secondary injuries. Nursing interventions such as skin hydration, use of emollients, reduction in friction forces, repositioning, and application of protective barriers proved effective in preventing these conditions. The discussion emphasizes that systematic nursing assessment and protocol-based care significantly contribute to reducing dermatological complications and improving care quality. The review concludes that evidence-based nursing practices are essential for promoting skin health in older adults and preventing avoidable injuries.	04
Spin M et al. “Skin tears in the elderly.” Estima–Brazilian Journal of Enterostomal Therapy, 2021; 19. DOI: 10.30886/estima.v19.1002_IN [42]	To identify in the scientific literature the knowledge produced about skin tears injuries in the elderly. Methods: An integrative literature review (2014–2019), carried out by searching the databases/platforms National Library of Medicine, Biomedical Answers and Biblioteca Virtual em Saúde, with descriptors and the Boolean operators “and” and “or“. Results: From the bibliographic search, selection and analysis, eight articles made up the sample. For skin tears injuries in the elderly, four pillars of care emerged: maintenance of organic and tissue homeostasis with a focus on proper nutrition and hydration; avoid trauma to senile skin, providing a safe environment with suitable devices; and the systematization of health care and education for elderly skin care. As for prevention mechanisms, primary prevention is achieved through a unique care plan and health education activities, focused on risk factors and vulnerabilities, minimizing damage and complications.	02
Jiang Q et al. “Knowledge status of skin tear prevention and its demographic and occupational influencing factors: A national cross-sectional survey among nurses.” Journal of Advanced Nursing, 2024. DOI: 10.1111/jan.16353 [43]	A skin tear (ST) is a common skin injury that is often misdiagnosed or overlooked. This study examined the current level of nurses’ knowledge about STs and the factors that influence it. Study design: A national cross-sectional survey combined with quantitative analysis was used to provide evidence of low knowledge levels among nurses regarding skin tear management and its influencing factors. Methods: An electronic questionnaire survey was conducted with 1293 nurses from 32 hospitals across 18 provinces in China. The survey included a General Information Questionnaire, the Nursing Knowledge Assessment Instrument (OASES), and the Self-Directed Learning Competence Scale for Nurses (SLCS-N). Results: The mean OASES score was 9.51 ± 3.15, with a correct response rate of 47.55%. Pearson correlation analysis showed positive correlations ranging from none to strong among all OASES dimensions, and strong to extremely strong correlations among all SLCS-N dimensions. Multivariate analysis identified several independent factors influencing knowledge related to wound/ostomy/incontinence care, including hospital level, presence of specialized wound/ostomy/incontinence nurses, participation in related training programs, willingness to attend ostomy-care training, self-evaluation of ostomy-care knowledge, and the degree of emphasis placed by managers. Conclusion: Overall knowledge about skin tear management was low among nurses nationwide. Nursing managers should establish comprehensive and specialized curricula, develop standardized and evidence-based care processes, and provide personalized training programs tailored to nurses’ unique characteristics and individual needs, thereby improving their proficiency in knowledge and skills related to skin tear prevention and management.	02
Hu R et al. “The skin tears knowledge among geriatric ward nurses and associated factors: A cross-sectional study.” Journal of Tissue Viability, 2024. DOI: 10.1016/j.jtv.2024.07.015 [44]	To determine the level of knowledge about skin tears among nurses working in geriatric wards and to identify associated factors. Methods: This cross-sectional study was conducted in southwestern China with 1172 nurses from geriatric wards in 10 hospitals. Data were collected through the Chinese online platform Sojump, and the Skin Tear Knowledge Assessment Instrument was used to evaluate participants’ knowledge. The analysis included descriptive statistics, correlation analysis, and multiple linear regression. Results: Participants had a mean age of 36.73 years (SD = 6.54). More than half had less than 10 years of experience in geriatric wards. A total of 27% were wound-care specialists, and 68.1% had no specific training in skin tears (STs). Additionally, 82.7% of geriatric nurses had never been exposed to guidelines on ST prevention and management. In geriatric wards, 36.6% of nurses had received training on ST prevention. The mean knowledge score was 9.52 (SD = 2.39) out of 18 points. The ‘Treatment’ domain had the lowest mean score, while ‘Specific patient groups’ had the highest. Multiple linear regression revealed that nurses’ knowledge of skin tears was influenced by sex (β = 0.096, *p* < 0.001), educational level (β = 0.062, *p* < 0.001), participation in ST-related training (β = −0.193, *p* < 0.001), participation in wound-care training (β = −0.120, *p* = 0.004), and specialization as a wound-care nurse (β = −0.350, *p* = 0.001). These factors explained 61.3% of the variance in knowledge scores. Conclusion: Geriatric ward nurses demonstrated limited knowledge about skin tears. To improve their skills in managing these injuries, nursing managers should provide targeted training and establish standardized, evidence-based care processes.	02
LeBlanc K, Woo KY, Van Den Kerkhof E, Woodbury MG. “Risk Factors Associated with Skin Tear Development in the Canadian Long-term Care Population.” Adv Skin Wound Care. 2021; 34(2): 87–95. doi: 10.1097/01.ASW.0000717232.03041.69. [45]	To examine the risk factors associated with the development of skin tears among older adults living in long-term care facilities in Ontario. Methods: A prospective study was conducted to explore risk factors associated with the development of skin tears (STs). A total of 380 individuals aged 65 years or older from four long-term care facilities in Ontario were assessed for ST at baseline and again at week four to determine incident cases. Researchers created an ‘ST table’ to record demographic characteristics, clinical variables, and skin assessments. Results: The study found a baseline prevalence of skin tears of 20.8% and an incidence of 18.9%. Baseline history of skin tears (risk ratio [RR] 1.84; 95% confidence interval [CI] 1.25–2.70; *p* = 0.002); presence of age-related skin changes such as ecchymosis and bruising (RR 1.60; 95% CI 1.43–1.79; *p* < 0.001); chronic disease (RR 1.17; 95% CI 1.03–1.32; *p* = 0.018); need for assistance with activities of daily living (RR 1.13; 95% CI 1.08–1.18; *p* < 0.001); and aggressive behavior (RR 1.06; 95% CI 1.02–1.10; *p* = 0.001) were significant risk factors associated with the development of ST. Conclusions: These findings provide much-needed data from Ontario on risk factors associated with the development of skin tears and can support prevention programs aimed at reducing ST risk in long-term care settings.	02
Yuceler Kacmaz H et al. “Skin tears in older patients in intensive care units: A multicentre point prevalence study.” Nursing in Critical Care, 2024; 1. DOI: 10.1111/nicc.13131 [46]	To determine the prevalence of pressure injuries and associated factors among elderly patients hospitalized in ICUs. Study design: This regional, multicenter point-prevalence study was conducted in five centers located in the five most populous cities of the Central Anatolia region in Turkey. Data were collected simultaneously in all centers on the same day. A list of ICU patients present on the day of data collection was generated, and 200 patients aged 65 years or older who agreed to participate were included. Researchers created an ‘ST table’ to record demographic characteristics, clinical variables, and skin assessments. Results: Pressure injuries (PI) were detected in 14.5% of ICU patients, with 72.5% presenting stage 1 PI. Significant associations were found between PI occurrence and mean body mass index (BMI) (*p* = 0.043), age (*p* = 0.014), and length of ICU stay (*p* = 0.004). Skin temperature (*p* = 0.002) and skin turgor (*p* = 0.001) were also significantly associated with PI, with higher prevalence observed in patients with cold skin and reduced turgor. PI prevalence was higher among individuals with a previous history of PI. Additionally, significant associations were found between PI and level of consciousness (*p* = 0.014), incontinence (*p* = 0.006), Braden score (*p* = 0.004), and Itaki fall-risk score (*p* = 0.006). Conclusion: In this multicenter point-prevalence study, the prevalence of pressure injuries among elderly ICU patients was 14.5%, and multiple associated factors were identified.	02

**Table 2:** Suggestions and changes made to the “Friction Care” game content.

Judges’ Suggestions	Changes Implemented
View: Risk factors and clinical assessment	Clinical assessment and identification of risk factors for FI
Review: Degree of FI	Category of friction injury type
Add: Porous regenerating membrane Membracel, barrier spray, acrylic foam	Added
Include a link to return to the questions	Return to previous question
I forgot my password and was unable to recover it	The item “change password” was added.

**Table 3:** Results of the Content Validity Coefficient based on the analysis conducted by the specialists. Pouso Alegre (MG), Brazil. 2025.

Evaluated Questions	Content Validity Coefficient	
	First Evaluation %	Second Evaluation %
Friction Care game content evaluation		
Definition of friction injury	0.99	0.99
Clinical assessment and identification of risk factors for friction injury in patients	0.94	1.0
Cleaning of friction injury	1.0	1.0
Category I of friction injury	0.95	1.0
Category II of friction injury	0.94	1.0
Category III of friction injury	0.94	1.0
Preventive measures for friction injury	0.97	1.0
Description of treatment according to the classification of friction injury	0.98	1.0
Friction Care Game functionality evaluation		
The Friction Care game accurately performs its functions.	1.0	1.0
Does the content present relevant information for the target audience?	1.0	1.0
The Friction Care game provides the necessary functions to assess, prevent, and treat friction injury.	1.0	1.0
The Friction Care game ensures secure access through password use.	0.98	1.0
Friction Care Game usability evaluation		
It is easy to understand the instructions of the Friction Care game.	1.0	1.0
When playing Friction Care, does the content facilitate the teaching and learning process on the topic?	1.0	1.0
It is easy to learn how to use the Friction Care game.	1.0	1.0
Players have full control over actions and can easily undo errors.	1.0	1.0
The tutorial is clear and objective, helping to clarify doubts effectively.	1.0	1.0
Friction Care Game efficiency evaluation		
Is the vocabulary accessible to the target audience?	1.0	1.0
The response time of the Friction Care game is adequate.	1.0	1.0
During the game, players remain motivated and wish to continue playing.	1.0	1.0
The stability, loading speed, and overall performance of the game are consistent.	1.0	1.0
The resources available in the Friction Care game are adequate.	1.0	1.0

**Table 4:** Judges’ evaluation of the Friction Care game content using the Delphi Technique. Pouso Alegre (MG), Brazil. 2025.

Evaluated Topics	First Evaluation				Second Evaluation			
	IND	PAD	ADQ	TAD	IND	PAD	ADQ	TAD
	n (%)	n (%)	n (%)	n (%)	n (%)	n (%)	n (%)	n (%)
Friction Care game content evaluation								
Definition of friction injury	0 (0)	04 (08)	34 (68)	12 (24)	0 (0)	1 (02)	32 (64)	17 (34)
Clinical assessment and identification of risk factors for friction injury in patients	01 (02)	02 (04)	34 (68)	13 (26)	0 (0)	0 (0)	35 (70)	15 (30)
Cleaning of friction injury	0 (0)	01 (02)	36 (72)	13 (26)	0 (0)	0 (0)	38 (76)	12 (24)
Category I of friction injury	01 (02)	01 (02)	34 (68)	14 (28)	0 (0)	0 (0)	36 (72)	14 (28)
Category II of friction injury	01 (02)	02 (04)	34 (68)	13 (26)	0 (0)	0 (0)	34 (68)	16 (32)
Category III of friction injury	0 (0)	01 (02)	35 (70)	14 (28)	0 (0)	0 (0)	33 (66)	17 (34)
Preventive measures for friction injury	0 (0)	03 (06)	39 (78)	08 (16)	0 (0)	0 (0)	34 (68)	16 (32)
Treatment according to the classification of friction injury	0 (0)	01 (02)	38 (76)	11 (22)	0 (0)	0 (0)	36 (72)	14 (28)
Friction Care Game functionality evaluation								
The Friction Care game accurately performs its functions.	0 (0)	01 (02)	33 (66)	16 (32)	0 (0)	0 (0)	40 (80)	10 (20)
Does the content present relevant information for the target audience?	0 (0)	0 (0)	35 (70)	15 (30)	0 (0)	0 (0)	41 (82)	09 (18)
The Friction Care game provides the necessary functions to assess, prevent, and treat friction injury.	0 (0)	0 (0)	35 (70)	15 (30)	0 (0)	0 (0)	38 (76)	12 (24)
The Friction Care game ensures secure access through password use.	0 (0)	1 (02)	36 (72)	13 (26)	0 (0)	0 (0)	37 (74)	13 (26)
Friction Care Game usability evaluation								
It is easy to understand the instructions of the Friction Care game.	0 (0)	0 (0)	41 (82)	09 (18)	0 (0)	0 (0)	39 (78)	11 (22)
When playing Friction Care, does the content facilitate the teaching and learning process on the topic?	0 (0)	0 (0)	38 (76)	12 (24)	0 (0)	0 (0)	38 (68)	12 (24)
It is easy to learn how to use the Friction Care game.	0 (0)	0 (0)	35 (70)	15 (30)	0 (0)	0 (0)	41 (82)	09 (18)
Players have full control over actions and can easily undo errors.	0 (0)	01 (02)	38 (76)	11 (22)	0 (0)	0 (0)	34 (68)	16 (32)
The tutorial is clear and objective, helping to clarify doubts effectively.	0 (0)	0 (0)	40 (80)	10 (20)	0 (0)	0 (0)	28 (56)	22 (44)
Friction Care Game efficiency evaluation								
Is the vocabulary accessible to the target audience?	0 (0)	0 (0)	37 (74)	13 (26)	0 (0)	0 (0)	34 (68)	16 (32)
The response time of the Friction Care game is adequate.	0 (0)	0 (0)	37 (74)	13 (26	0 (0)	0 (0)	33 (66)	17 (34)
During the game, players remain motivated and wish to continue playing.	0 (0)	0 (0)	42 (84)	08 (16)	0 (0)	0 (0)	36 (72)	14 (28)
The stability, loading speed, and overall performance of the game are consistent.	0 (0)	0 (0)	36 (72)	14 (28)	0 (0)	0 (0)	38 (76)	12 (24)
The resources available in the Friction Care game are adequate.	0 (0)	01 (02)	36 (72)	13 (265)	0 (0)	0 (0)	39 (78)	11 (22)

IND—inadequate; PAD—partially adequate; ADQ—adequate; TAD—totally adequate.

## Data Availability

The data supporting the findings of this study are not publicly available due to ethical and privacy restrictions. However, the statistical analyses used in this study are archived in the institution’s statistics department and may be requested from the responsible staff member at victors@univas.edu.br.

## Public Involvement Statement

No members of the public were involved in the design, conduct, or interpretation of this study. Participation was limited to volunteers who completed the questionnaires, without additional involvement in the development or review of the results.

## Guidelines and Standards Statement

This study was conducted in full compliance with the ethical and regulatory requirements applicable to research involving human participants, including the Declaration of Helsinki and the standards established by the Research Ethics Committee of the University of Vale do Sapucaí. All procedures were carried out in accordance with recognized scientific and ethical standards.

## Abbreviations

The following abbreviations are used in this manuscript:

CVC	Content Validity Coefficient
FI	Friction Injury
FICT	Free and Informed Consent Term

## Appendix A. Examples of Questions Answered by Users During Gameplay

1. What is a friction injury?

( ) Injury caused by separation of the skin layers due to friction

( ) Injury caused exclusively by pressure

( ) Injury caused by thermal burn

( ) Injury caused by chemical agents

2. Which skin structure is most frequently affected in a friction injury?

( ) Hypodermis

( ) Muscle tissue

( ) Epidermis and dermis

( ) Bone tissue

3. A friction injury occurs mainly when:

( ) There is direct impact

( ) There is prolonged sun exposure

( ) The skin slides against a surface

( ) There is contact with irritating substances

4. A friction injury is characterized by:

( ) Partial or total separation of the skin layers

( ) Total rupture of the skin

( ) Deep necrosis

( ) Primary bacterial infection

5. What is the internationally used term for friction injury?

( ) Skin tear

( ) Burn injury

( ) Pressure ulcer

( ) Abrasion wound

6. Type 1 friction injury is characterized by:

( ) Total loss of skin

( ) Intact skin without separation

( ) Separated skin but with a viable flap

( ) Infected injury

7. Type 2 friction injury presents:

( ) Non-viable flap

( ) Partial loss of skin

( ) Total loss of skin

( ) Viable flap that cannot be repositioned

8. Type 3 friction injury corresponds to:

( ) Total loss of skin

( ) Viable flap

( ) Superficial lesion without bleeding

( ) Injury caused by impact

9. The classification of friction injury is based on:

( ) Depth of the injury

( ) Skin color

( ) Flap viability and skin loss

( ) Presence of pain

10. Which classification system is widely used for friction injury?

( ) Braden

( ) ISTAP

( ) Norton

( ) Glasgow

11. An essential measure to prevent friction injury is:

( ) Use of hot water for hygiene

( ) Vigorously rubbing the skin

( ) Applying ice daily

( ) Keeping the skin moisturized

12. To reduce the risk of friction injury, it is recommended to:

( ) Remove adhesives quickly

( ) Use protective barriers on the skin

( ) Avoid using moisturizers

( ) Keep the skin dry and rough

13. A factor that increases the risk of friction injury is:

( ) Intact skin

( ) Use of soft clothing

( ) Dry, fragile skin

( ) Good nutrition

14. When mobilizing the patient, one should:

( ) Use techniques that avoid friction

( ) Drag the body across the sheet

( ) Pull the patient by the arms

( ) Avoid using assistive devices

15. In the treatment of Type 1 friction injury, the flap should be:

( ) Removed

( ) Gently repositioned

( ) Dried with gauze

( ) Rubbed to make it adhere

16. Which product is indicated to protect the skin around a friction injury?

( ) Alcohol

( ) Hydrogen peroxide

( ) Barrier spray

( ) Scented soap

17. A porous regenerative membrane (e.g., Membracel) is used to:

( ) Protect and promote healing

( ) Remove devitalized tissue

( ) Dry the lesion

( ) Reduce the skin temperature

18. In Type 3 friction injury, it is usually necessary to:

( ) Reposition the flap

( ) Apply ice

( ) Remove non-viable tissue and protect the area

( ) Use rigid adhesives

19. Cleansing of a friction injury should be performed with:

( ) Normal saline

( ) Hot water

( ) Scented soap

( ) Irritating solutions

20. Which type of dressing is most indicated when the skin flap is viable and repositioned?

( ) Rigid adhesive dressing

( ) Non-adherent dressing

( ) Dry gauze

( ) Compression dressing

21. In friction injuries, non-adherent dressings are preferred because they:

( ) Increase traction on the flap

( ) Reduce skin hydration

( ) Prevent the dressing from sticking to the skin and causing a new injury

( ) Promote chemical debridement

22. In friction injuries, why are dressings with silicone borders recommended?

( ) Because they adhere strongly to the skin

( ) Because they speed up mechanical debridement

( ) Because they increase pressure on the flap

( ) Because they reduce the risk of new injuries when removed
